# Alzheimer's Type Neuropathological Changes in a Patient with Depression and Anxiety: A Case Report and Literature Review of Neuropathological Correlates of Neuropsychiatric Symptoms in Alzheimer's Disease

**DOI:** 10.1155/2023/5581288

**Published:** 2023-10-11

**Authors:** Sumit Das

**Affiliations:** ^1^Department of Laboratory Medicine and Pathology (Neuropathology), University of Alberta Hospital, Edmonton, Alberta, Canada; ^2^Neuroscience and Mental Health Institute, University of Alberta, Edmonton, Alberta, Canada

## Abstract

Alzheimer's disease (AD) is classified as a tauopathy and is the most common neuropathological correlate of dementia/cognitive impairment. AD is neuropathologically characterized by the presence of beta-amyloid immunoreactive senile plaques and tau positive neurofibrillary tangles. Neuropsychiatric symptoms of AD however continue to be underscored, and therefore, neuropathological correlates of these neuropsychiatric symptoms are not readily studied. Presented here is a case of 60-year-old female who initially presented with anxiety and depression, and continued to be the predominant symptoms although mild cognitive impairment was noted as per the available clinical notes. Postmortem examination of the brain revealed severe Alzheimer's type neuropathological changes, which included significant tau and beta-amyloid pathology in limbic regions, which were thought to represent correlates of the patient's depression and anxiety. This case report illustrates the possible neuropathological correlates of neuropsychiatric symptoms in patients with AD. The author hopes that such a case will promote more in-depth studies into the pathophysiology of neuropsychiatric manifestations in AD.

## 1. Introduction

Alzheimer's disease (AD) is regarded as a tauopathy associated with cognitive and functional decline in typically elderly (>65 years) patients although cases of early-onset (before age of 65 years) are possible [1]. Neuropathological correlates of AD include beta-amyloid immunoreactive senile plaques and tau immunoreactive neurofibrillary tangles. The neuropathological staging of Alzheimer's type neuropathological changes is a proposed method for predicting the probability that these changes are related to the presentation of dementia/cognitive decline [2]. What is not accounted for in the staging system are the noncognitive symptoms such as neuropsychiatric signs and symptoms. Neuropsychiatric symptoms are common in MCI reported to occur in 35–75% of patients. The most common of these symptoms include apathy, anxiety, depression, irritability, and agitation. These behavioural symptoms have been thought to help identify patients with mild cognitive impairment suspected of presenting with prodromal AD [3]. Presented here is a case of neuropathologically confirmed AD where the predominant symptoms were anxiety and depression. This case represents the only known case to present the possible neuropathological correlates of the patient's neuropsychiatric symptoms.

## 2. Case Report

Limited clinical information was available on the electronic medical chart. As per the information available, this female patient who was 60 years-old at first presentation reported difficulty with short-term memory along with difficulty sleeping. Psychiatric history included depression and anxiety. Neurological examination was notable for mini mental status (MMSE) of 25/30. The patient was clinically diagnosed with mild cognitive impairment based on the MMSE score. Unfortunately, no additional clinical information regarding her cognition could be found. With regards to her depression and anxiety, the patient reported feeling sad about some aspects of her life, and the episodes of sadness were exacerbated by difficulty in her first marriage and from having to switch employment. Clinically, she was diagnosed with major depressive disorder, generalized anxiety disorder, and mild cognitive decline.

The patient passed away at hospice facility. After passing, a restricted autopsy limited to postmortem examination of the brain was requested to determine the etiology of her cognitive impairment.

The weight of the unfixed brain was recorded at 1330 g. The cerebral hemispheres did not seem to display any significant atrophy. The coronal slices of the cerebral hemispheres did not reveal evidence of atrophy. Ventricular caliber appeared unremarkable. Hippocampus and amygdala did not appear shrunken. Pigment was visible in both the substantia nigra of the midbrain and locus ceruleus of the pons.

**Figure 1:**
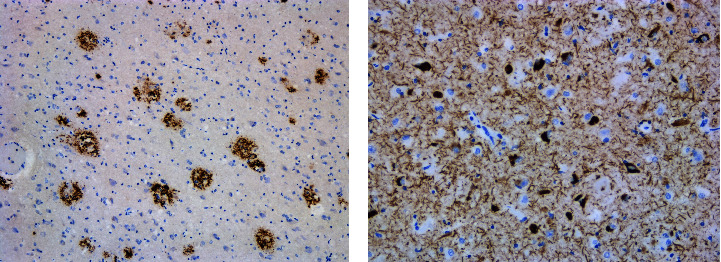
(a) Beta-amyloid immunohistochemistry on the amygdala revealing senile plaques; (b) tau immunohistochemistry on the amygdala revealing NFTs, neurites, and neuropil threads.

Histologic examination with immunohistochemical work-up revealed evidence of cerebral amyloid angiopathy in the form of thickened blood vessels in the leptomeninges that were stained by beta amyloid immunohistochemistry. Sampled sections from the cerebral hemispheres showed several beta amyloid positive diffuse plaques and moderate amount of senile plaques throughout neocortex, including cingulate, calcarine, and entorhinal cortices, as well as in the thalamus. There were also scattered tau positive neurofibrillary tangles, neurites, and neuropil threads throughout the cortical gray matter including the superficial and deep layers of the calcarine cortex and entorhinal cortex. Thickened blood vessels that were not stained by beta amyloid immunohistochemistry, and hence consistent with arteriolosclerosis, were noted in the subcortical white matter. Left amygdala showed scattered tau positive neurofibrillary tangles, pretangles, neurites, neuropil threads, and argyrophilic grains. There were also numerous beta-amyloid positive diffuse and senile plaques in the amygdala. Examples of these findings are shown in Figure 1. The left and right hippocampus showed tau positive neurofibrillary tangles, neurites, and neuropil threads predominantly throughout the CA2 and CA1 sectors of the hippocampus and less frequently in the CA3 and CA4 sectors.

Midbrain showed some beta amyloid positive diffuse and senile plaques. Although beta amyloid staining was observed in blood vessels of the cerebellar leptomeninges, no definite beta amyloid staining is seen within the cerebellar parenchyma.

The final neuropathological diagnosis was reported as Alzheimer's type neuropathological changes (amyloid deposition score = A3 (Thal phase 4); Braak (neurofibrillary tangle) stage = 6 of 6; CERAD (senile plaque) score = C2 (moderate)); cerebral amyloid angiopathy; relatively mild arteriolosclerosis.

## 3. Discussion

To the author's knowledge, this case is one of the first case examples to demonstrate neuropathological correlation between Alzheimer's type neuropathological changes and clinical history of neuropsychiatric symptoms. Neuropsychiatric symptoms are described in patients with Alzheimer's disease, and animal models have suggested that behavioural and psychological symptoms increase with progressive neuropathology [4]. Depression and anxiety, as noted in our patient, have been suggested to correlate with atrophy of frontal, temporal, and insular areas [5], although in our patient, no significant cerebral atrophy could be detected. The contribution of neuropsychiatric symptoms to cognitive decline has also been studied by some. Fuller et al. suggested in their analysis of demographic, neurocognitive, neuroimaging, and neuropsychiatric symptom data from their AD neuroimaging initiative database (*n* = 906) that psychotic symptoms (hallucinations and delusions) were more strongly related to the continuum of AD-associated cognitive dysfunction than other neuropsychiatric symptoms. The group also observed a negative correlation between psychotic symptoms and brain volume [6]. Based on these studies, our case seems unusual in the sense that our patient's symptoms consisted primarily of anxiety and depression, while cognitive impairment seemed to be mild and a minor component of her clinical history. This would be considered discrepant with the high degree of Alzheimer's type neuropathological changes observed in our patient. Based on the findings in this case, the author believes the high amyloid content in limbic regions such as the amygdala, cingulate, and entorhinal areas may be the main neuropathological correlates of our patient's neuropsychiatric manifestations. It is difficult to be certain as to the reason behind the lack of cognitive dysfunction noted given the advanced Alzheimer's pathology. Aside from the limited clinical information available, one possibility may be the severity of the neuropsychiatric symptoms that may have masked any degree of cognitive impairment. Neuropathological data to explain neuropsychiatric symptoms are scarce, and therefore, the precise mechanism behind neuropsychiatric symptoms in AD remains unclear. One of the rare studies includes work by Gibson et al. whose group reviewed postmortem neuropathological cases from 2004 to 2021 (*n* = 1038) that included AD and Lewy body disease pathology. The authors reported higher neurofibrillary tangle (NFT) stages to be associated with depression and agitation and hallucinations associated with higher Lewy body stages [7]. These results would seem to be consistent with the findings in our patient, which also exhibited high NFT stage. A systematic review by Botto et al. that involved a review of 14760 studies and 34 papers on AD patients suggested that neurodegeneration of areas and circuits dealing with emotions can elicit the anxious and depressive symptoms that, in turn, can lead to further neurodegeneration. The authors also suggested the severity of anxiety and depression is greater in early-onset AD [8].

Interestingly, genetic etiologies shared between AD and depression have been reported. Chiba‐Falek et al. performed pleiotropy analysis using genome-wide association studies (GWAS) from publicly accessible websites of AD GWAS and major depressive disorder (MDD) GWAS. The group found moderate enrichment of single nucleotide polymorphism (SNP) associated with late-onset AD (LOAD) across increasingly stringent levels of significance with the MDD GWAS association (LOAD|MDD), of maximum four and eight-folds, including and excluding the *APOE*-region, respectively. Association analysis excluding the *APOE*-region identified numerous SNPs corresponding to 40 genes, 9 of which were known LOAD-risk loci primarily in chromosome 11 regions containing the *SPI1*gene and *MS4A* genes cluster, and others were novel pleiotropic risk-loci for LOAD conditional with MDD [9]. Genetic testing was not available for our patient.

Herein, the author has presented neuropathologically severe Alzheimer's type neuropathological changes in a patient whose predominant presentation seems to be neuropsychiatric in the form of anxiety and depression. It is hoped that such a case will encourage further studies of the neuropathological correlates and pathophysiology of neuropsychiatric symptom in AD and other neurodegenerative disorders.

## Data Availability

No data were used to support the findings of this study.
